# Synthesis and Assessment of Antibacterial Activities of Ruthenium(III) Mixed Ligand Complexes Containing 1,10-Phenanthroline and Guanide

**DOI:** 10.1155/2016/3607924

**Published:** 2016-10-19

**Authors:** Atakilt Abebe, Tizazu Hailemariam

**Affiliations:** Science College, Bahir Dar University, Bahir Dar, Ethiopia

## Abstract

In this work, two complexes of ruthenium(III) ([Ru(phen)_2_Cl_2_]Cl·2H_2_O and [Ru(phen)_2_(G)Cl]2Cl·H_2_O) were synthesized from 1,10-phenanthroline alone as well as from both 1,10-phenanthroline and guanide. The synthesis was checked using halide test, conductance measurement, and spectroscopic (ICP-OES, FTIR, and UV/Vis) analysis. Their in vitro antibacterial activities were also investigated on two Gram-positive (*Staphylococcus aureus* (*S. aureus*) and methicillin resistant* Staphylococcus aureus* (MRSA)) and two Gram-negative (*Escherichia coli* (*E. coli*) and* Klebsiella pneumoniae* (*K. pneumoniae*)) bacteria. These complexes showed wide-range better activities than the commercially available controls (Chloramphenicol and Ciprofloxacin) against even the most drug resistant* K. pneumoniae*. [Ru(phen)_2_(G)Cl]2Cl·H_2_O inhibited* S. aureus*, MRSA,* E. coli*, and* K. pneumoniae* by 17.5%, 27.4%, 16%, and 52%, respectively, better than Chloramphenicol. It also inhibited these pathogens by 5.9%, 5.1%, 2.3%, and 17.2%, respectively, better than Ciprofloxacin. Similarly, [Ru(Phen)_2_(Cl)_2_]Cl·2H_2_O inhibited these pathogens by 11%, 8.7%, 0.1%, and 31.2%, respectively, better than Chloramphenicol. Therefore, after in vivo cytotoxicity investigations, these compounds can be considered as potential antibiotic drugs.

## 1. Introduction

Coordination chemistry is about tuning properties of metal ions using different ligands [1–3]. This includes stabilization of different oxidation states and modulation of the solvophilicity and electrophilic and nucleophilic properties of the metal ion [4–6]. While coordinating, the properties of the ligands themselves are also modified. For instance, the pharmacological activities and their crucial role in DNA/RNA base pairing through several hydrogen-bonding patterns of free oxypurines such as guanine can significantly change after complex formation [7, 8]. Based on this, synthesis of different coordination compounds with desired properties by ligand tailoring has become a fascinating research field. Designing new coordination compounds with therapeutic abilities has been part of this activity [9–17]. From this perspective, there has been a growing interest in the chemistry community to examine the biological activities of ruthenium complexes [18, 19]. Ruthenium (5*s* ^*2*^4*d* ^*6*^) frequently accesses +2 and +3 oxidation states at physiological conditions and can interact with nucleic acids, proteins, sulfur, or oxygen containing compounds and water in the cells [20–24]. Its interaction kinetics with the latter can be controlled using advantages of the unique properties of each kind of ligand. This enables the ligand exchange rates of ruthenium complexes to be close to those of cellular processes which make them suitable for therapeutic applications.

In this respect, numerous investigations in the properties and applications of ruthenium(II) complexes with 1,10-phenanthroline (phen) as a ligand or mixed with other ligands are reported [25–27]. Nevertheless, there is no report on the chemistry of ruthenium(III) complex containing 1,10-phenanthroline alone or 1,10-phenanthroline and guanide mixed.

The ideally placed nitrogen atoms along with their rigid planar structure and hydrophobic, electron-poor heteroaromatic, and *π*-acidic properties cooperatively made 1,10-phenanthroline a classic chelating bidentate ligand. These properties enable it to have stacking interaction ability with DNA base pairs [28–31]. Guanine is a chemically inert oxypurine heteroaromatic molecule. Its inertness is changed by complex formation. The latter is favored in its guanide (G^−^) form which is derived by deprotonation of guanine.

The purpose of this study is to examine the effects of 1,10-phenanthroline alone and mixed with guanide on the biological activity of Ru(III). The complex would orchestrate the binding ability of ruthenium with a range of biomolecules, the unique stacking interaction ability of 1,10-phenanthroline on cell genetic material, and the interaction of guanide through hydrogen bonding with cytosine residue of the genetic material.

## 2. Experimental

### 2.1. Chemicals

All chemicals used in the present work are as follows: 1,10-phenanthroline monohydrate (BDH Chemical Ltd., Poole, England), guanine (99%, ACROS), RuCl_3_, silver nitrate, sodium hydroxide, acetone, chloroform, sulfuric acid (Sigma Aldrich), methanol (Hi Media Laboratories Ltd., India), KBr, dichloromethane, Mueller Hinton agar, and barium chloride (BLULUX Laboratories Ltd., India), and nitric acid (T.V. Industrial Estate, India).

### 2.2. Instruments and Methods

The electronic conductance was measured using 10^−3 ^M solution of each complex in deionized water with JENWAY 4200 conductivity meter at room temperature. The electronic spectra were recorded in the 200–800 nm region on Sanyo SP65 UV/Vis spectrophotometer. IR spectra were recorded using KBr discs in the 4000–400 cm^−1^ region on AVATAR 330 FTIR, Thermo Nicolet spectrophotometer. Ruthenium content was determined by PerkinElmer, Optima 7300 V HF Version ICP-OES spectrometer, digesting 10 mg of each complex in concentrated nitric acid and diluting them using distilled water. Melting points were determined using STONE, STAFFORDSHIRE, ST15 OSA, UK, digital melting point apparatus. Chloride ions were determined thermogravimetrically using the AgCl precipitate obtained from the mixture of 10 mL solution of 1 mg of each complex in distilled water with excess AgNO_3_ solution.

### 2.3. Synthesis

#### 2.3.1. Synthesis of [Ru(Phen)_2_(Cl)_2_]Cl·2H_2_O

Ethanolic solution of 1,10-phenanthroline (1 g, 5 mmol) was added from a dropping funnel to an ethanolic solution of RuCl_3_ (0.5 g, 2.5 mmol) being stirred magnetically in an ice bath. The mixture was allowed to stir for 3 h at room temperature. Reddish-brown homogeneous solution was obtained. The solvent was removed in vacuum. Reddish-brown powder was collected and washed three times with acetone to remove any unreacted 1,10-phenanthroline. It was recrystallized from ethanol to remove any unreacted RuCl_3_ (yield: 1.2 g, 80%).

#### 2.3.2. Synthesis of [Ru(Phen)_2_(G)(Cl)]Cl·H_2_O

**Scheme 1:**
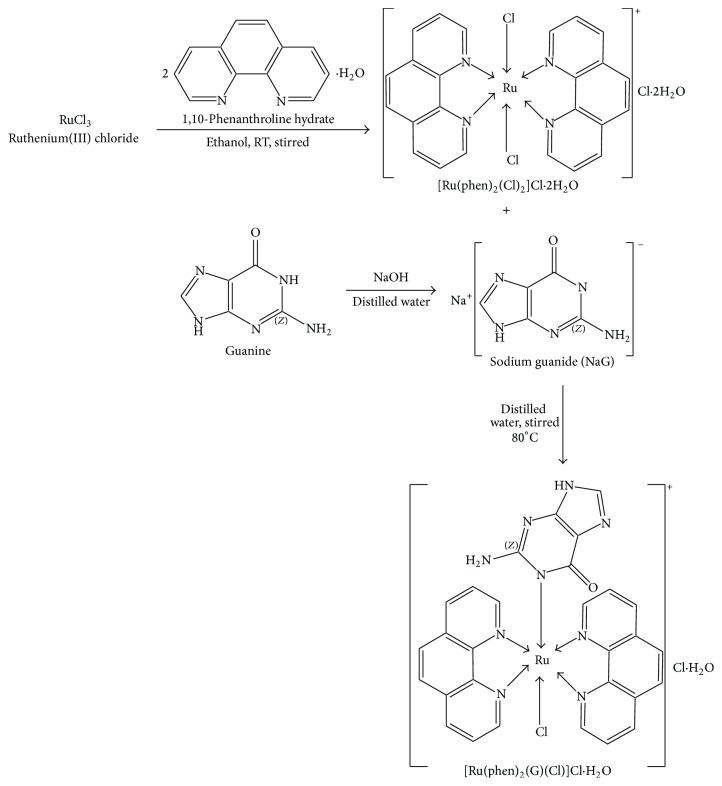
Synthesis of [Ru(phen)_2_(Cl)_2_]Cl·2H_2_O and [Ru(Phen)_2_(G)(Cl)]Cl·2H_2_O.

An aqueous solution of sodium guanide obtained from a reaction between guanine (0.125 g, 8.0 mmol) and sodium hydroxide (0.032 g, 8.0 mmol) was added from a dropping funnel to an aqueous solution of [Ru(Phen)_2_(Cl)_2_]Cl·2H_2_O (0.5 g, 8.0 mmol) while stirring in an oil bath at 80°C. The mixture was allowed to stir for 3 h. The resulting orange-red homogeneous solution was mixed with 100 mL dichloromethane and stirred for 1 h and allowed to stand overnight. The organic (dichloromethane) phase was separated using separatory funnel. The dichloromethane was removed in vacuum and dark brown powder was collected and recrystallized from ethanol (yield: 0.546 g, 87%). The reaction paths of the two synthetic complexes are shown in Scheme 1.

### 2.4. Antibacterial Activity Testing

The ligands and their metal complexes were evaluated for in vitro antibacterial activities against strains of two Gram-positive (*S. aureus* (ATCC 25923) and methicillin resistant* S. aureus* (clinical isolate)) and two Gram-negative (*E. coli* (ATCC255922) and* K. pneumoniae* (ATCC986605)) bacteria. The bacterial strains were maintained in the appropriate blood agar base at 4°C. Antibiotic discs (Ciprofloxacin 5 *μ*g and Chloramphenicol 30 *μ*g) were used as reference. The minimum inhibitory concentration (MIC) against each bacterium was determined by preparing aqueous solutions of different concentrations of the complexes by serial dilution (200 *μ*g/mL, 300 *μ*g/mL, 400 *μ*g/mL, 500 *μ*g/mL, 600 *μ*g/mL, 800 *μ*g/mL, and 1000 *μ*g/mL). The experiments were repeated three times to obtain consistent results. The antibacterial tests were carried out at the Amhara Regional Health Research Microbiology Laboratory Center, Bahir Dar, Ethiopia.

## 3. Results and Discussion

The analytical data of the complexes are indicated in Table 1.

### 3.1. Molar Conductance of the Metal Complexes

The conductance measurements, recorded for 10^−3 ^M solutions of the metal complexes in deionized water, are listed in Table 1. The data shows that both complexes are 1 : 1 electrolytes [32]. The lower conductance of [Ru(phen)_2_(G)(Cl)]Cl·H_2_O compared to [Ru(phen)_2_Cl_2_]Cl·2H_2_O is a consequence of increase in molar mass and the surface area. Hence the speed of mobility of the cation decreases as a result of the decrease in the kinetic energy imparted by the electric field from measurement instrument [33].

### 3.2. Electronic Spectra

**Figure 1:**
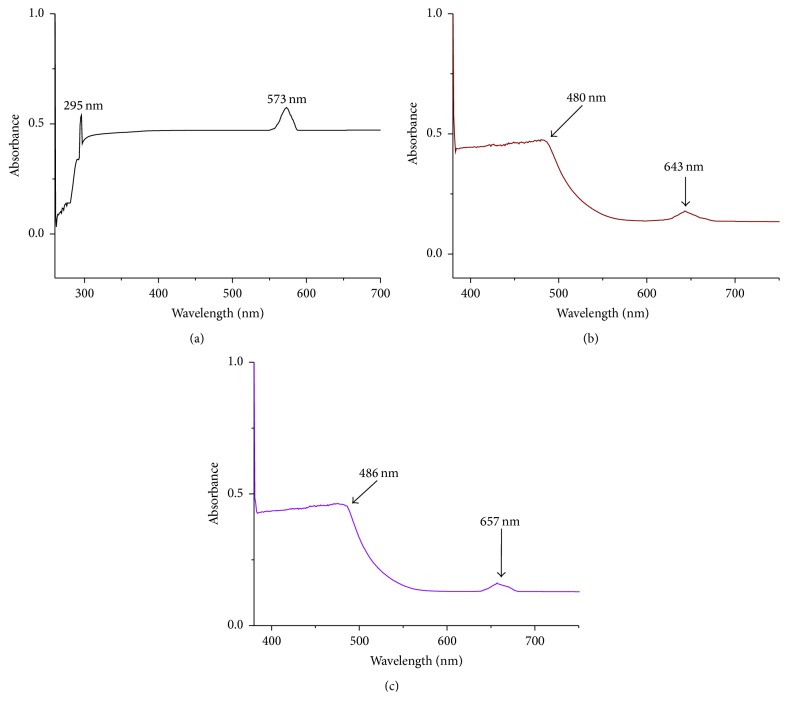
Electronic spectra of (a) RuCl_3_, (b) [Ru(phen)_2_Cl_2_]Cl·2H_2_O, and (c) [Ru(phen)_2_(G)Cl]Cl·H_2_O.

The electronic spectra of the complexes are displayed in Figure 1 and Table 2.

The complexes exhibited simple characteristic d-d transitions. The difference in the band position for d-d transition absorption of RuCl_3_ and the complexes may be explained by assuming different environments around the metal ion following the coordination [34, 35]. The coordination of 1,10-phenanthroline to Ru(III) results in a distorted octahedral geometry. Consequently, the *e*_g_ orbitals split to d_*z*2_ and d_*x*2-*y*2_ resulting in two transitions (t_2g_ → *e*_g_(d_*z*2_) and t_2g_ → *e*_g_(d_*x*2-*y*2_)). [Ru(Phen)_2_(G)(Cl)]Cl·H_2_O demonstrated absorption at longer wavelength (Figure 1(c)) than [Ru(Phen)_2_(Cl)_2_]Cl·2H_2_O (Figure 1(b)). This is probably because guanide (G^−^) formed a shorter and stronger bond that narrowed the transition (t_2g_ → *e*_g_) gap (Figure 1 and Table 2).

### 3.3. IR Spectroscopy

**Figure 2:**
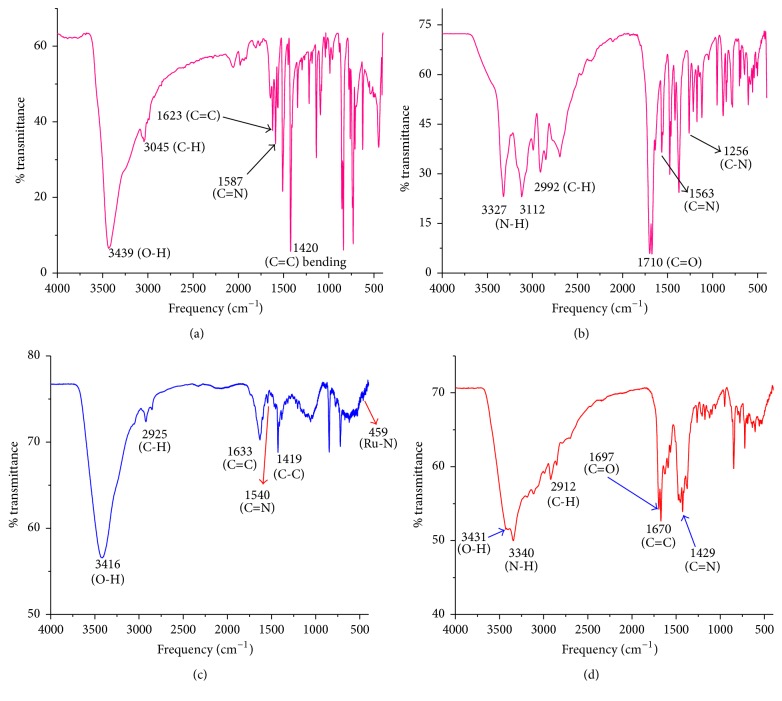
FTIR spectra of (a) 1,10-phenanthroline monohydrate, (b) guanine, (c) [Ru(phen)_2_(Cl)_2_]Cl·2H_2_O, and (d) [Ru(phen)_2_(G)(Cl)]Cl·H_2_O.

The infrared spectra of the ligands and the complexes are indicated in Figure 2 and selected characteristic frequencies are indicated in Table 3.

The bands at 1623 cm^−1^ (s) and 1587 cm^−1^ (s), characteristic for *ν*_C=C_ and *ν*_C=N_ stretching in the free 1,10-phenanthroline monohydrate (Figure 2(a)), appear at 1670 cm^−1^ (w) and 1429 cm^−1^ (w), respectively, in [Ru(phen)_2_(G)(Cl)]Cl·H_2_O (Figure 2(d)). They also appeared at 1633 cm^−1^ (w) and 1540 cm^−1^ (w), respectively, in [Ru(phen)_2_(Cl)_2_]Cl·2H_2_O (Figure 2(c)). Similarly, the characteristic bands of guanide at 3335 cm^−1^ (s), 3112 cm^−1^ (s) *ν*_N-H_(NH_2_), and 1710 cm^−1^ (s) (*ν*_C=O_) (Figure 2(b)) are displaced towards 3340 cm^−1^ (w) and 1697 cm^−1^ (w) (Figure 2(d)), respectively. The changes in absorption frequencies and strength suggest that 1,10-phenanthroline and guanide are coordinated. The strong and broad bands at 3439 cm^−1^ and 3416 cm^−1^ characteristic for *ν*_O-H_(H_2_O) in the free 1,10-phenanthroline monohydrate and [Ru(phen)_2_(Cl)_2_]Cl·2H_2_O, respectively (Figures 2(a) and 2(b)), appear at 3431 cm^−1^ obscured in the band characteristic for *ν*_N-H_(NH_2_) in [Ru(phen)_2_(G)(Cl)]Cl·H_2_O (Figure 2(d)). This change in the absorption frequency of water explains the change in the nature of its interaction. Moreover, the change in the intensity may explain the change in the relative amount of water molecules in 1,10-phenanthroline monohydrate and the complexes. The latter argument supports the proposed formula of the complexes.

### 3.4. Antibacterial Activity Testing

**Figure 3:**
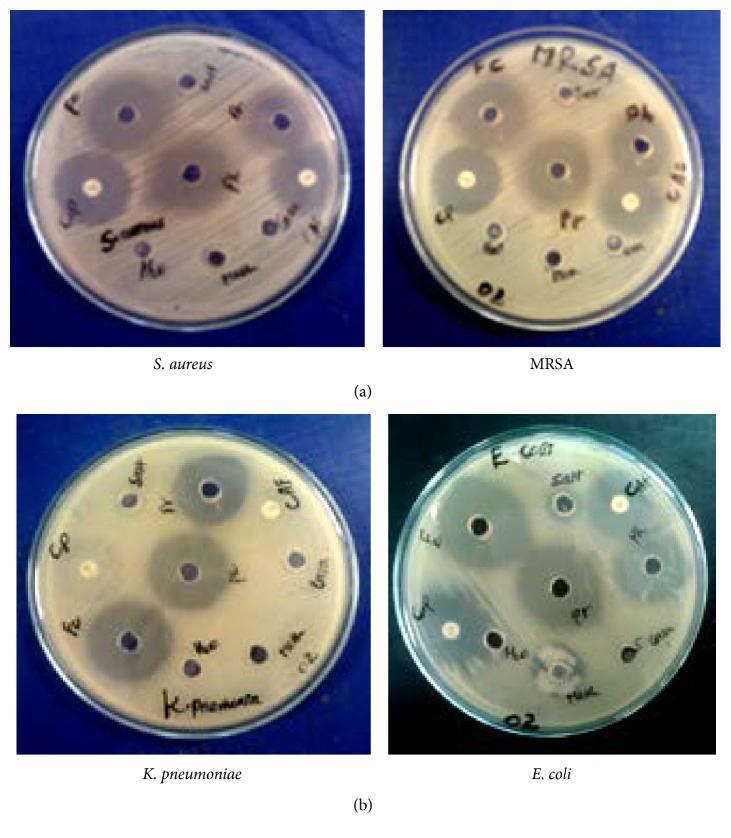
Inhibition zone of ligands, complexes, salt, and commercial antibiotics against (a) Gram-positive (*S*.* aureus* and MARSA) and (b) Gram-negative (*K*.* pneumoniae* and* E*.* coli*) bacteria.

This observation shows that the complexes demonstrated biological activities against all the tested strains (Figure 3 and Table 4). The observed increase in antibacterial activity can be explained on the basis of Overtone's concept [36] and Tweedy's chelation theory [37]. The lipid membrane that surrounds the cell favors the passage of only lipid soluble materials which is an important condition for antimicrobial activity. On coordination, the polarity of the metal ion will be reduced to a greater extent due to the overlap of the ligand orbitals and partial sharing of the positive charge of the metal ion with the donor groups. Further, it increases the delocalization of *π* electrons over the whole chelate ring and hence enhances the liposolubility of the complexes. This increased liposolubility enhances the penetration of the complexes into the lipid membrane and interferes in the normal activities of the bacteria [38].

The percent activity indexes of the complexes against the reference antibiotics demonstrated significant comparative inhibitions (Table 5). [Ru(phen)_2_(G)(Cl)]Cl·H_2_O showed better activity than the two commercial antibiotics (Ciprofloxacin and Chloramphenicol) against all the strains studied including the most drug resistant Gram-negative* K*.* pneumoniae*. [Ru(phen)_2_(Cl)_2_]Cl·2H_2_O also showed nearly equal activities as Chloramphenicol against* S. aureus* and* E. coli* and better activities against MARSA and* K*.* pneumoniae* (Table 5). The better activities demonstrated by [Ru(phen)_2_(G)(Cl)]Cl·H_2_O compared to [Ru(phen)_2_(Cl)_2_]Cl·2H_2_O are due to its additional interaction with the genetic material of the cell by guanide.

### 3.5. Minimum Inhibitory Concentration (MIC) Determination

MIC is the lowest concentration that completely inhibited the growth of microorganisms for 24 hours.

Around 300 *μ*g/mL [Ru(Phen)_2_(G)(Cl)]Cl·H_2_O is sufficient to inhibit the growth of* E. coli* while around 400 *μ*g/mL is necessary to start inhibiting* S. aureus* and* K. pneumonia* (Table 6).

## 4. Conclusions

In this synthesis, Ru(III) and the ligands are brought together with rigid configuration. This resulted in delocalization of *π* electrons over the whole cationic unit and hence the reduction of the polarity of the complexes which increased the liposolubility. This has enhanced the penetration of the complexes into the lipid membrane and inhibited the growth of the tested Gram-positive and Gram-negative bacteria. The latter phenomenon demonstrates the wide-range activities of the complexes.

## Figures and Tables

**Table 1:** Analytical data of the complexes.

Complex (color)	Melting point/°C	Yield (%)	Elemental estimation Calculated (found) (%)		Molar conductivity Λ_M_ (S cm^2^ mol^−1^)
			Ru	Cl	
[Ru(phen)_2_(Cl)_2_]Cl·2H_2_O (reddish brown)	>300	80	16.74 (16.62)	5.88 (5.66)	121.50
[Ru(phen)_2_(G)(Cl)]Cl·H_2_O (orange red)	>300	87	14.47 (14.23)	5.07 (4.89)	87.26

**Table 2:** Electronic spectral data of the salt and complexes.

Complex	Band position (nm)	Assignment
RuCl_3_	295, 573	^*∗*^LMCT, d-d(^2^T_2g_ → ^2^T_1g_)
[Ru(phen)_2_(Cl)_2_]Cl·2H_2_O	643	d-d(^2^T_2g_ → ^2^T_1g_)
[Ru(phen)_2_(G)(Cl)]Cl·H_2_O	657	d-d(^2^T_2g_ → ^2^T_1g_)

^*∗*^LMCT: ligand to metal charge transfer.

**Table 3:** Important characteristic IR bands of the ligands and complexes, cm^−1^.

Compound	Absorption frequencies, cm^−1^						
	*ν*(O-H)	*ν*(N-H)	*ν*(C-H)	*ν*(C-N)	*ν*(C=C)	*ν*(C=N)	*ν*(C=O)
1,10-Phenanthroline monohydrate	3439 (s, b)	—	3045 (w)	1290 (w)	1623 (s)	1587 (s)	—
Guanine	—	3335, 3112 (d)	2992	1256 (w)	1692	1563 (w)	1710 (s)
[Ru(Phen)_2_(Cl)_2_]Cl·2H_2_O	3416 (s, b)	—	2925 (w)	1208 (w)	1633 (w)	1540 (w)	—
[Ru(Phen)_2_(G)(Cl)]Cl·H_2_O	3431	3340 (s)	2912 (w)	1270	1670	1429	1697

s: strong, b: broad, w: weak, and d: doublet.

**Table 4:** Antibacterial activities of the complexes, free ligands, metal salt, and commercially available antibiotics.

Compound	Inhibition zone (mm)			
	*S. aureus *(ATCC 25923)	MRSA (clinical isolate)	*E. coli* (ATC 255922)	*K. pneumonia *(ATCC 986605)
1,10-Phenanthroline	27.40 ± 0.12	26.4 ± 0.11	28.20 ± 0.12	26.00 ± 0.14
Guanine	—	—	—	—
RuCl_3_	—	—	12.20 ± 0.21	—
[Ru(Phen)_2_(Cl)_2_]Cl·2H_2_O	25.54 ± 0.15	24.56 ± 0.2	26.53 ± 0.13	26.50 ± 0.13
[Ru(Phen)_2_(G)(Cl)]Cl·H_2_O	29.65 ± 0.13	28.8 ± 0.21	30.70 ± 0.11	30.70 ± 0.11
Ciprofloxacin	28.00 ± 0.14	27.4 ± 0.13	30.00 ± 0.10	26.20 ± 0.32
Chloramphenicol	25.24 ± 0.14	22.6 ± 0.15	26.50 ± 0.23	20.20 ± 0.35

**(a):** 

Microorganism				
Compound	*S. aureus*	MRSA	*E. coli*	*K. pneumoniae*
[Ru(Phen)_2_(Cl)_2_]Cl·H_2_O	11.00%	8.70%	0.10%	31.20%
[Ru(Phen)_2_(G)(Cl)]Cl·H_2_O	17.50%	27.40%	15.85%	52.00%

**(b):** 

Microorganism				
Compound	*S. aureus*	MRSA	*E. coli*	*K. pneumoniae*
[Ru(Phen)_2_(Cl)_2_]Cl·2H_2_O	−8.80%	−10.00%	−12.00%	1.01%
[Ru(Phen)_2_(G)(Cl)]Cl·H_2_O	5.90%	5.10%	2.30%	17.17%

MRSA: methicillin resistant *S*. *aureus*.

**Table 6:** MIC assay of [Ru(Phen)_2_(G)(Cl)]Cl_2_·H_2_O against four bacterial pathogens.

Microorganism	Minimum concentration of microorganism growth						
	200 *µ*g/mL	300 *µ*g/mL	400 *µ*g/mL	500 *µ*g/mL	600 *µ*g/mL	800 *µ*g/mL	1000 *µ*g/mL
*S. aureus*	**+**	**+**	**−**	**−**	**−**	**−**	**−**
MRSA	**+**	**+**	**+**	**+**	**−**	**−**	**−**
*K. pneumoniae*	**+**	**+**	**−**	**−**	**−**	**−**	**−**
*E. coli*	**+**	**−**	**−**	**−**	**−**	**−**	**−**

Note: +: bacteria growth and −: no bacteria growth.
